# Draft Genome Sequence of the Cellulolytic Bacterium *Clostridium papyrosolvens* C7 (ATCC 700395)

**DOI:** 10.1128/genomeA.00698-13

**Published:** 2013-09-12

**Authors:** Veronica Zepeda, Bareket Dassa, Ilya Borovok, Raphael Lamed, Edward A. Bayer, Jamie H. D. Cate

**Affiliations:** Department of Molecular and Cell Biology, University of California, Berkeley, California, USAa; Department of Biological Chemistry, the Weizmann Institute of Science, Rehovot, Israelb; Department of Molecular Microbiology and Biotechnology, Tel Aviv University, Ramat Aviv, Israelc; Department of Chemistry, University of California, Berkeley, California, USAd; Physical Biosciences Division, Lawrence Berkeley National Laboratory, Berkeley, California, USAe

## Abstract

We report the draft genome sequence of the cellulose-degrading bacterium *Clostridium papyrosolvens* C7, originally isolated from mud collected below a freshwater pond in Massachusetts. This Gram-positive bacterium grows in a mesophilic anaerobic environment with filter paper as the only carbon source, and it has a simple cellulosome system with multiple carbohydrate-degrading enzymes.

## GENOME ANNOUNCEMENT

The microbial degradation of plant cell walls is a key step in nature that contributes to the global carbon cycle (1). Many types of bacteria and fungi utilize the cellulose in plant cell walls as their primary energy and carbon source (2, 3). Some organisms use large multienzyme complexes called cellulosomes to efficiently depolymerize crystalline cellulose (4, 5). Cellulosomes are assembled as extracellular complexes where multiple degradative enzymes are bound to a scaffolding protein, known as the scaffoldin. Enzymes dock onto the scaffoldin through noncatalytic dockerin modules, which bind cognate modules on the scaffoldins called cohesins. The scaffoldin subunit also contains a carbohydrate binding module (CBM) that attaches the entire enzymatic complex to the cellulose substrate. Cellulosomes differ between organisms, particularly in their number of cohesin modules and specificity of the dockerin-cohesin interactions (6). The scaffoldin subunit also contains a single CBM that attaches the entire enzymatic complex to the cellulose substrate.

*Clostridium papyrosolvens* strain C7 is a Gram-positive bacterium isolated from mud collected below a freshwater pond in Massachusetts (7). It was one of eight strains selected for its ability to grow in a mesophilic anaerobic environment with filter paper as the only carbon source.

Strain C7 was grown at 37°C on MJ minimal medium, with Avicel as the sole carbon source, according to previously described methods (8). High-molecular-weight genomic DNA was isolated using cetyltrimethylammonium bromide and was sequenced by 454 Life Sciences, yielding 20× coverage and 104 large contigs (>500 bp) containing 4.4 Mb of DNA. An analysis of all the data (1,671 contigs, 4.7 Mb) led to the prediction of 4,061 gene models containing open reading frames (ORFs), which is similar to the number observed in other cellulolytic clostridia. Phylogenetic analysis of the conserved genes has shown that strain C7 is closely related to but distinct from the previously sequenced *C. papyrosolvens* strain DSM 2782 (9).

A reciprocal BLAST search of this database against the 3,639 *Clostridium cellulolyticum* H10 predicted genes found that 2,596 (64%) of the strain C7 gene models are putative orthologs. Further analysis identified CpC7_725 as the ORF encoding a single scaffoldin for incorporating dockerin-containing enzymes. The 137-kDa scaffoldin protein contains one N-terminal CBM domain, six cohesins, and three domains of unknown function, commonly referred to as χ^2^ domains. The C7 genome encodes 72 dockerin-containing proteins that can be incorporated into cellulosomes via the cohesin-bearing scaffoldin subunit.

C7 contains a cellulosome-related gene cluster, known to be an essential part of active cellulosomes (10), which is identical in gene order to *C. cellulolyticum* H10 (ORFs CpC7_725 to CpC7_736). The C7 cellulosome can thus be classified as a simple cellulosome system versus the more complex systems, such as those of *Clostridium thermocellum* and related species (11, 12). However, elements of the scaffoldin operon promoter region contain insertions, deletions, or are highly variable compared to that of *C. cellulolyticum* H10. Thus, the regulation of the *cel* operon and other gene clusters in C7 might differ substantially from what has been observed in related species.

### Nucleotide sequence accession number.

The draft genome sequence is deposited at DDBJ/EMBL/GenBank under the accession no. ATAY00000000.
